# Acute dyspnea in the emergency department: a clinical review

**DOI:** 10.1007/s11739-023-03322-8

**Published:** 2023-06-02

**Authors:** Pierachille Santus, Dejan Radovanovic, Marina Saad, Camilla Zilianti, Silvia Coppola, Davide Alberto Chiumello, Matteo Pecchiari

**Affiliations:** 1 Division of Respiratory Diseases, Ospedale Luigi Sacco, Polo Universitario, ASST Fatebenefratelli-Sacco, Via G.B. Grassi 74, 20157 Milan, Italy; 2 Department of Biomedical and Clinical Sciences (DIBIC), Università Degli Studi Di Milano, Milan, Italy; 3 Department of Pathophysiology and Transplantation, Università Degli Studi Di Milano, Milan, Italy; 4 Department of Anesthesia and Intensive Care, ASST Santi Paolo E Carlo, Ospedale Universitario San Paolo, Milan, Italy; 5 Department of Health Sciences, Università Degli Studi Di Milano, Milan, Italy; 6 Coordinated Research Center On Respiratory Failure, Università Degli Studi Di Milano, Milan, Italy

**Keywords:** Dyspnea, Respiratory failure, Respiratory support, Non-invasive ventilation, High flow oxygen, Cardiogenic edema

## Abstract

Acute dyspnea represents one of the most frequent symptoms leading to emergency room evaluation. Its significant prognostic value warrants a careful evaluation. The differential diagnosis of dyspnea is complex due to the lack of specificity and the loose association between its intensity and the severity of the underlying pathological condition. The initial assessment of dyspnea calls for prompt diagnostic evaluation and identification of optimal monitoring strategy and provides information useful to allocate the patient to the most appropriate setting of care. In recent years, accumulating evidence indicated that lung ultrasound, along with echocardiography, represents the first rapid and non-invasive line of assessment that accurately differentiates heart, lung or extra-pulmonary involvement in patients with dyspnea. Moreover, non-invasive respiratory support modalities such as high-flow nasal oxygen and continuous positive airway pressure have aroused major clinical interest, in light of their efficacy and practicality to treat patients with dyspnea requiring ventilatory support, without using invasive mechanical ventilation. This clinical review is focused on the pathophysiology of acute dyspnea, on its clinical presentation and evaluation, including ultrasound-based diagnostic workup, and on available non-invasive modalities of respiratory support that may be required in patients with acute dyspnea secondary or associated with respiratory failure.

## Introduction

Dyspnea is a broad term indicating a variety of unpleasant sensations related to breathing, including air hunger (the sensation which can be elicited by prolonging a breath-hold), sense of work/effort, or of chest tightness [1, 2]. Dyspnea can vary in intensity and quality depending on the underlying pathophysiological mechanism, and its perception is influenced by the patient’s social, cultural and psychological characteristics [3]. Acute dyspnea is one of the main reasons for admission to the emergency department (ED) [4], as it is common in a variety of diseases, including cardiorespiratory, infectious and oncologic diseases [5].

Its intensity on arrival at the ED predicts hospital admission [6], and it is associated with a long length of stay in ED [7] together with a high in-hospital fatality rate [7, 8]. Physicians need to make a rapid diagnosis and choose a treatment plan based on limited clinical information [9]. Since accurate and rapid management can be lifesaving [10], making a differential diagnosis in patients with acute dyspnea in the ED is a clinical challenge requiring complex decision-making to decrease hospital mortality and the length of stay [10].

The primary aim of this review is to recall the principal pathophysiology patterns of dyspnea, as well as the diagnostic strategy and therapeutic management of acute dyspnea in an emergency setting, focusing on non-invasive ventilation.

## Pathophysiology

The present discussion will focus on the elementary mechanisms that, usually in combination, trigger dyspnea in patients accessing the ED. For a more detailed and systematic discussion of the pathophysiology of dyspnea, excellent reviews are available [3, 11, 12].

### Hypercapnia/hypoxia

Contrary to early belief that acute hypercapnia is unable to elicit dyspnea directly, but only through reflex changes in respiratory activity [13], studies on healthy subjects have shown that an increase of arterial pressure of carbon dioxide (PaCO_2_) by itself can produce breathlessness [14]. Mild hypoxia slightly enhances hypercapnia-induced air hunger, while the act of breathing markedly reduces it, in line with previous studies on breath-hold [15, 16].

An acute increase of PaCO_2_, potentially contributing to dyspnea, is a common occurrence in many diseases, including acute respiratory distress syndrome (ARDS) [17], chronic obstructive pulmonary disease (COPD) and asthma during exacerbations [18, 19], and acute heart failure [20].

In addition, acute hypoxia can induce air hunger, but the decrease of arterial pressure of oxygen (PaO_2_) necessary to trigger an unpleasant respiratory sensation is much bigger than the 4 mmHg increase of PaCO_2_ required to elicit the same effect, at least in healthy subjects. When ventilation is constrained, healthy subjects in normocapnic conditions perceive air hunger only when PaO_2_ falls below ~ 60 mmHg [21].

### Mechanical loading

A sense of increased work of breathing/effort can be evoked in a variety of situations in which the elastic or resistive load against which the respiratory muscles contract increases, or in which the ability of the respiratory muscles to generate pressure is impaired. A decrease in respiratory muscle performance may arise both because of the weakness of the respiratory muscles or because the respiratory muscles have to contract in a situation of mechanical disadvantage, due to a change of their operating length, as in the presence of static or dynamic hyperinflation [22, 23].

In clinical settings, a common cause of dynamic hyperinflation is the occurrence of tidal expiratory flow-limitation (EFL), that is the inability of expiratory flow to increase in response to an increase of driving pressure at isovolume [24]. During breathing at rest, tidal EFL is often present in stable COPD patients [25, 26], and its occurrence increases markedly during exacerbations [27]. If hyperinflation is reduced by pharmacological interventions or by physical rehabilitation, dyspnea decreases [25, 28–30]. Tidal EFL is also frequently present in chronic heart failure patients in a supine position [31]. Orthopnea is more prominent in patients with tidal EFL, and the increase of dyspnea sensation during the postural change from standing to supine is greater in those patients showing a smaller increase or even a decrease of inspiratory capacity, suggesting that dynamic hyperinflation is a determinant of this perception [31]. During heart failure decompensation, tidal EFL may appear even in the standing position [32]. In this condition, acute treatment with vasodilators and diuretics may abolish tidal EFL and mitigate dyspnea sensation [33].

### Activation of pulmonary receptors

Indirect evidence suggests that information carried by vagal C and Aδ fibers plays a role in the genesis of the dyspnea perceived in pathological conditions characterized by pulmonary congestion, local mediators release or parenchymal alterations [34–38]. In pulmonary fibrosis, for instance, vagal afferents activation secondary to alveolitis has been proposed as a mechanism contributing to dyspnea [37, 38]. Particular attention should be paid to rapid onset dyspnea of unknown origin, as it is common in pulmonary thromboembolism (PE), where it can be the only symptom at rest or during exertion [39], and it often appears disproportionate to the level of blood gases alteration. Interestingly, activation of irritant receptors in asthma has been associated with the specific sensation of chest tightness or constriction, often experienced by these patients [37].

## Diagnostic evaluation

### Clinical presentation

Being exclusively self-reported, dyspnea should be assessed separately from signs indicating respiratory distress, such as tachypnea or accessory respiratory muscles activation, that may also be present independently of patients’ perception of breathlessness. In patients presenting with dyspnea, these signs, however, should be carefully evaluated, to increase the diagnostic accuracy of the underlying disease.

Ideally, the respiratory rate should be measured by observing the patient’s chest wall and abdominal movements for 60 s [40]. Resting respiratory rate in healthy adults spans between 12 and 22 breaths/min with no difference between young and old subjects [41], and increases to a variable extent in respiratory and non-respiratory diseases [42]. Indeed, the large range of normality of respiratory rate should be kept in mind when evaluating a patient in the acute care setting with the quickSOFA score, where a respiratory rate ≥ 22 breaths/min rises the risk of poor outcomes in patients with suspected sepsis [43]. Moreover, a high respiratory rate independently predicts the need for intubation [44]. Persistent tachypnea can be a sign of respiratory failure [45], neuromuscular diseases [46], acute pulmonary or extrapulmonary restrictive processes [42, 47], including pneumothorax [48], massive pleural effusion [49], or pain [50]. Rapid shallow breathing is difficult to assess in spontaneously breathing patients, therefore other signs such as “staccato speech” might be of help. To the opposite, increased tidal volumes with or without an increased breathing frequency (i.e. hyperpnea) can be secondary to metabolic acidosis in case of decompensated diabetes mellitus, renal failure or rhabdomyolysis.

Increased work of breathing is suggested by the recession of the suprasternal fossa and tracheal tug. Recession of the suprasternal fossa increases with increasing pleural pressure swings, often reflecting airway obstruction [51]. Tracheal tug is the manifestation of the downward motion of the trachea with each inspiratory effort and can be appreciated by inspecting and palpating the thyroid cartilage. It mirrors the dragging imposed by forceful diaphragm contraction on the entire mediastinum [51]. Inspection of intercostal spaces may reveal an inward motion of the lower ribcage during inspiratory efforts, the Hoover sign, indicating hyperinflation [52]. Other signs of increased work of breathing are represented by sternomastoid activation during inspiration (present in normal subjects only during strong inspiratory efforts [53], an occipital dorsi-flexion (reflecting trapezius contraction during inspiration), nasal flaring, mouth opening and inspiratory laryngeal groans.

### Blood biomarkers

Different blood biomarkers can help emergency physicians in making a differential diagnosis in patients with dyspnea, offering some additional information concerning its pathophysiology and suggesting further diagnostic evaluation.

#### Arterial blood gas analysis

Tensions and concentrations of O_2_ and CO_2_ constitute a mainstay of clinical care to assess the degree of pulmonary gas exchange abnormality [54]. The arterial blood gas analysis is a rapid diagnostic tool used to evaluate acute changes in blood pH, PaO_2_, and PaCO_2_. pH value at presentation is a predictor of short and long-term outcomes in acutely dyspneic patients presenting to ED. In this setting a pH level ≤ 7.39 is associated with a 37% mortality rate after 12 months [55].

From a diagnostic point of view, arterial blood gas analysis, including a PaO2 informative on gas exchange, should be always performed in patients with acute dyspnea in the ED. For monitoring purposes, in selected conditions, peripheral venous blood gas sampling may be a useful alternative, due to small differences between arterial and venous values for pH, and HCO_3_^−^ [56].

#### B-type natriuretic peptide

In acute conditions, plasma concentrations of natriuretic peptides (NPs) are recommended as initial diagnostic tests to differentiate cardiac from pulmonary causes of shortness of breath, in conjunction with other clinical and instrumental information.

In its guidelines, the European Society of Cardiology recommends threshold values of ≥ 100 pg/mL for B-type natriuretic peptide (BNP) and ≥ 300 pg/mL for N-terminal pro B-type natriuretic peptide (NT-proBNP), as lower values exclude acute heart failure [57]. However, it should be noted that many conditions can affect NPs plasma levels, reducing their diagnostic accuracy. Increasing age, acute or chronic kidney disease and atrial fibrillation tend to increase NPs levels, while obesity tends to reduce them [58].

#### Troponins and creatine kinase

In all patients presenting with acute dyspnea, especially when associated with chest pain, acute coronary syndrome (ACS) should be investigated. In this context, myocardial biomarkers, preferably high-sensitivity cardiac troponin (hs-cTn), are a useful complement to clinical assessment and 12-lead ECG in the diagnosis, risk stratification, and treatment of these patients [59].

However, hs-cTn present variable specificity for ACS, especially hs-cTnT, and may be elevated in the setting of PE, sepsis, pericarditis, myocarditis, warfarin use, and renal failure [60]. Performance of analytical assays has been shown to vary among manufacturers [60], and initial levels of these biomarkers measured at admission in the ED are frequently normal depending on the time from symptom onset [61]. However, when myocardial ischemia is suggested by the clinical presentation, then a rise of hs-cTn above the 99^th^ percentile of healthy individuals in serial sampling indicates myocardial infarction [62].

Measurement of cardiac troponins level is also important for risk stratification in patients with ACS, as higher concentrations are correlated with higher mortality [63].

#### D-dimer

D-dimer, a degradation product of cross-linked fibrin, increases in plasma following acute thrombus formation, because of simultaneous activation of coagulation and fibrinolysis; however, some other common pathological conditions often lead to high D-dimer levels, such as cancer, inflammation, and infections [64].

Patients at low risk for PE according to a validated scoring system (e.g., modified Wells criteria) and a negative D-dimer level, can be ruled out for PE without further testing [65]. In this regard, due to the decrease of D-dimer specificity with age, D-dimer cutoff should be adjusted for age in patients older than 50 years [66]. A positive result, however, only indicates the necessity of further diagnostics such as CT angiography which represents the main thoracic imaging test for a definite diagnosis when PE is suspected [67].

### Chest X-ray

Currently, a standard chest radiograph (CXR) is the first routine examination performed in patients presenting in the ED for dyspnea. It is considered the standard test for diagnosing pneumonia [68], even if false negatives may occur [69] and agreement in CXR interpretation between ED physicians and radiologists might be low [70]. CXR has been long considered the first-line diagnostic tool to be used in the diagnosis and quantification of pneumothorax [71] and pleural effusion [72]: orthostatic standard CXR in two projections is able to detect even a minimum amount of pleural effusion (about 50 mL), which is usually visualized at lateral projection only in the posterior costophrenic angle [73].

Pulmonary venous congestion, cardiomegaly, interstitial or alveolar edema (e.g., "Kerley B" lines, peribronchial cuffing), and pleural effusion suggest a cardiac origin of dyspnea; however, approximately 20% of patients admitted with heart failure have a non-diagnostic CXR, and other tools, such as cardiopulmonary ultrasound, can help in the diagnosis [74].

### Ultrasound

The utility of cardiac ultrasound in the ED is well-established and its use is the focus of many statements and guidelines [75, 76].

To the contrary, interest in lung ultrasound is more recent, as in the past ultrasonography was not considered an appropriate tool for lung imaging due to the presence of air, impeding proper visualization of pulmonary parenchyma. In the last three decades, however, a number of studies has uncovered several potentialities of this technique, and many ultrasound findings, previously considered artifacts without a precise meaning, are now signs which may help the diagnosis of critically ill patients [77, 78]. Since its introduction, lung ultrasound, in addition to echocardiography, proved to be at least as accurate as a standard of care in the assessment of several conditions presenting with acute dyspnea, such as pneumonia, pleural and pericardial effusion, pneumothorax and heart failure [79, 80]. Indeed, lung ultrasound showed higher sensitivity than CXR for free pleural effusion [80], and, according to one study, it has a high negative predictive value for heart failure, allowing to reliably rule out this disease as the main cause of dyspnea in ED patients [81]. Several studies reported that, compared with usual care, cardiopulmonary ultrasound had the advantage of a shorter delay to establish the etiology of acute dyspnea, with an overall high accuracy (> 90%) [80, 81]. Finally, the application of chest ultrasound for the triage of patients with dyspnea outside the hospital setting before or during transportation to the ED has increased intervention appropriateness and represents a promising tool also for trained nurses, medical technicians and paramedics in delivering pre-hospital diagnosis and care also outside the trauma setting [78, 80–82].

One of the most reliable protocols for the application of lung ultrasound in the emergency setting for the differential diagnosis of acute respiratory failure, and consequently of dyspnea, is the BLUE-protocol, developed by Lichtenstein in 2008 [83]. The protocol includes an assessment of the presence of "lung sliding" (a twinkling visible at the pleural line), "A-lines" (repetitive horizontal artifacts roughly parallel to the pleural line), and "B-lines" (a well-defined comet-tail artifact arising from the pleural line and erasing A-lines). According to the BLUE protocol, lung scans had to be bilateral, and acquired at three different points: a) the mid-sub-clavicular line, b) the point just crural to the nipple; c) at the point at which the posterior axillary line crosses the horizontal nipple line (PLAPS point–posterolateral alveolar and/or pleural Syndrome). The accuracy of the protocol in the identification of the cause of respiratory distress was very high with a sensitivity and a specificity between 90 and 100% in most cases [83].

"Lung sliding" together with "A lines" rules out the presence of interstitial edema (or interstitial syndrome, identified by the presence of vertical artifacts or “B lines”), lung consolidation (tissue-like ultrasound appearance of the lung), pleural effusion (presence of liquid between the lung and the parietal pleura) and pneumothorax (absence of lung sliding and presence of "lung point", an ultrasound image of the location at which separation by air between parietal and visceral pleura occurs). The presence of "lung sliding" and "A lines" in the absence of deep vein thrombosis excludes PE, while it may be suggestive of COPD or asthma exacerbation as a cause of dyspnea and respiratory failure after the exclusion of the previous diagnoses [83].

**Fig. 1:**
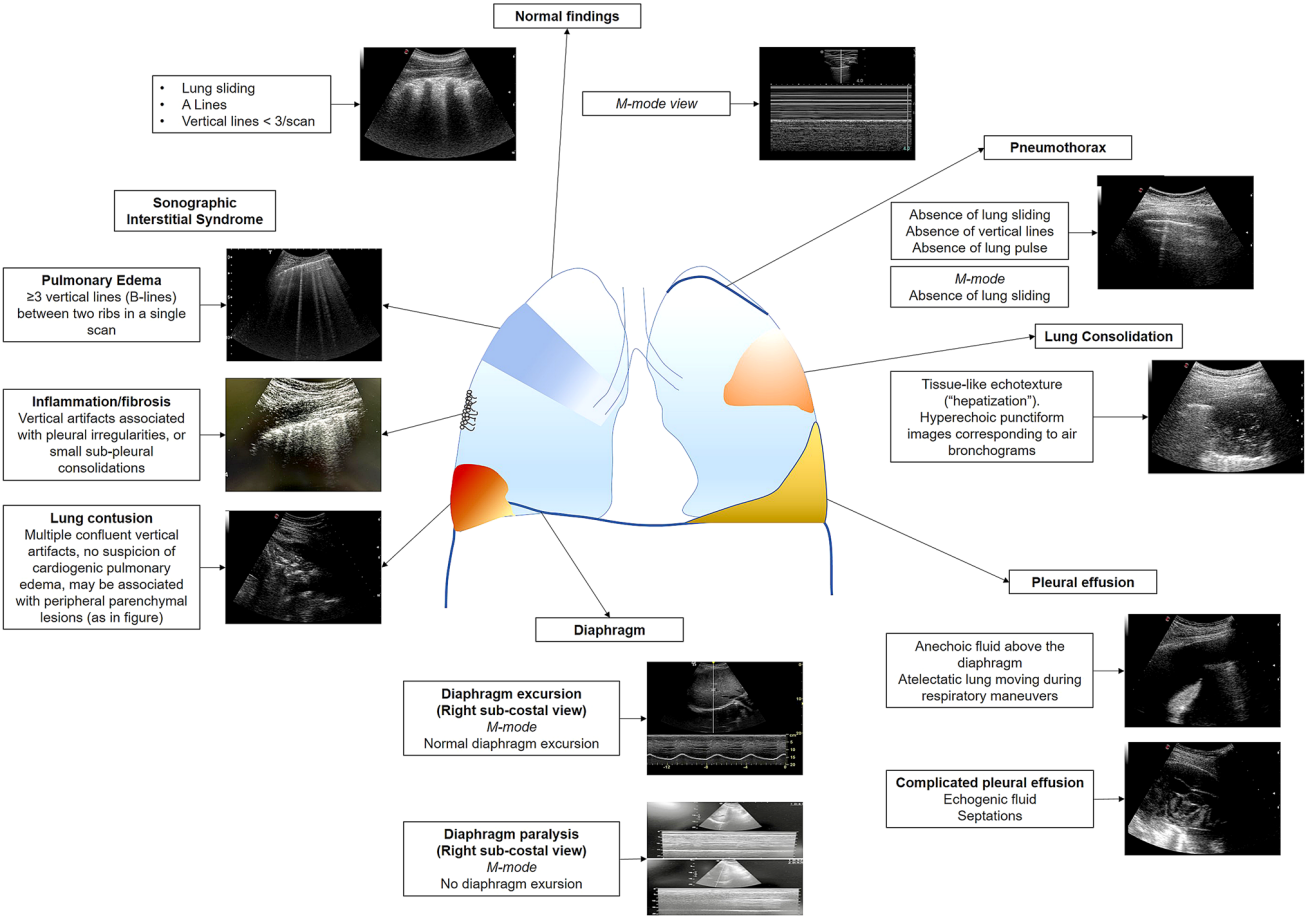
Main normal and pathological findings during lung ultrasound

In the last years, however, the evolving physical understanding of lung ultrasound is driving a re-evaluation of patterns such as the interstitial syndrome, owing to the fact that vertical artifacts (previously called “B lines”) have different characteristics in different chronic and acute diseases (e.g. lung fibrosis, interstitial edema, interstitial pneumonia, COVID-19 pneumonia, etc.) [77, 78, 84, 85]. A graphical overview of the main lung ultrasound findings is summarized in Fig. 1.

The information obtained by lung ultrasound should be integrated with that provided by echocardiography (as left ventricular ejection fraction (LVEF), right ventricular dimensions and cardiac wall motion abnormalities) and venous ultrasound to increase diagnostic accuracy and reliability.

Despite the numerous positive outcomes, lack of standardization, methodological issues and the consequent variability among studies in the true positives/true negatives rates of cardiopulmonary ultrasound in the assessment of various conditions [86] is probably the reason that led the American College of Physicians to prudently suggest bedside ultrasound just as a possible effective adjunctive tool for acute dyspnea assessment in ED [87]. However, as the literature on this topic expands, it is likely that, in the years to come, the use of ultrasound will become more and more widespread in the ED.

### Chest CT

Chest computer tomography (CT) scanning represents the gold standard for many pulmonary diseases, including PE and malignancy. Despite the emerging evidence on the usefulness of lung ultrasound, to date societal guidelines still do not recommend the use of sonography for the diagnosis of pneumonia or pneumothorax [87]; in these cases, CXR and CT scans are still considered more appropriate [88, 89].

Concerning PE, the lower sensitivity of the ultrasound diagnosis compared with that of the standard ED evaluation (40% vs 91%) is likely due to the essential information provided by the CT pulmonary angiography [81].

**Fig. 2:**
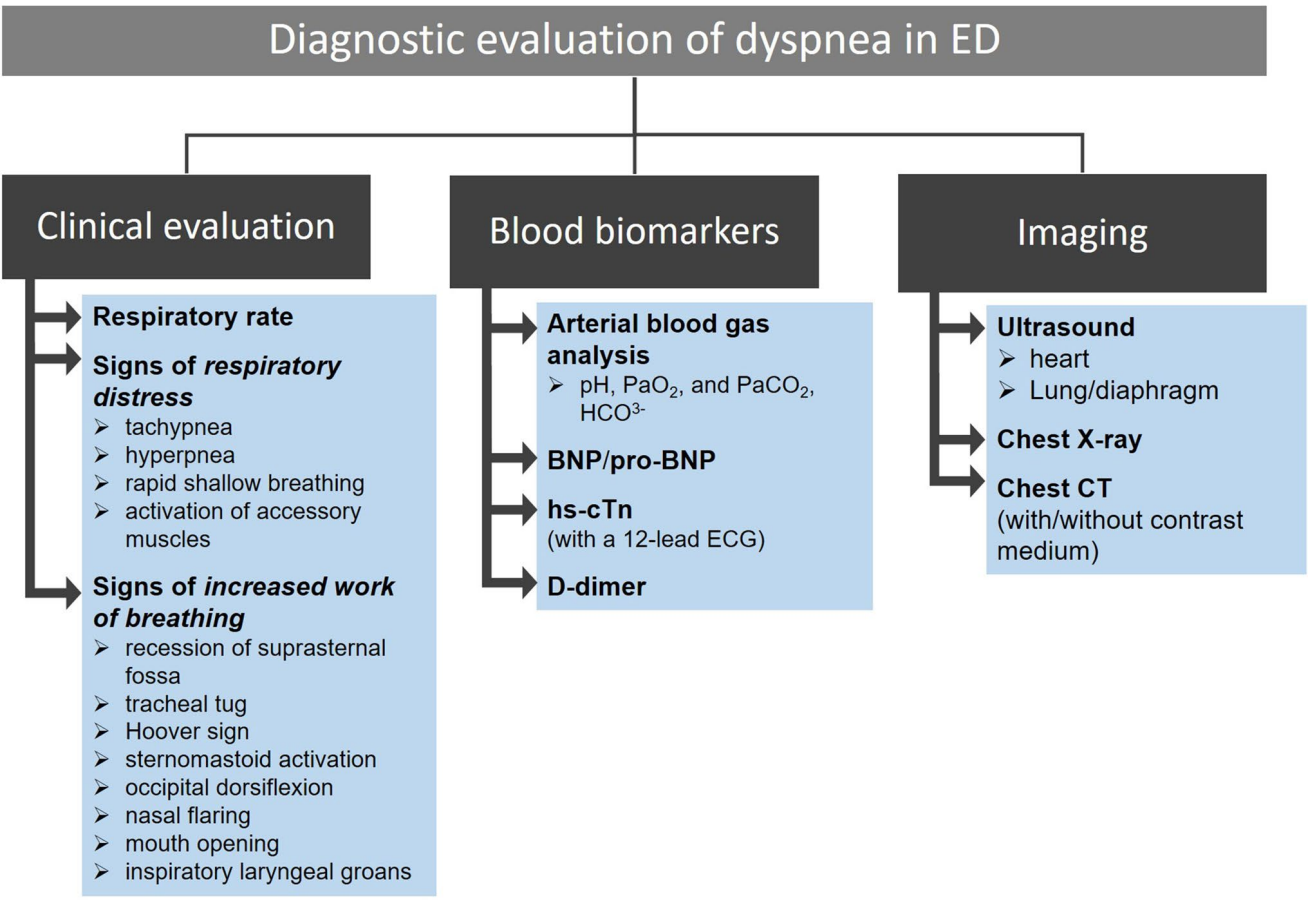
Summary of the diagnostic workup in patients with acute dyspnea presenting at the emergency department. BNP: B type natriuretic peptide; NT-proBNP: N-terminal pro B-type natriuretic peptide; hs-cTn: high sensitivity cardiac troponin; CT: computed tomography; PaO_2_: arterial partial pressure of oxygen; PaCO_2_: arterial partial pressure of carbon dioxide; HCO^3−^: bicarbonate

A diagram summarizing the diagnostic workup in patients with acute dyspnea is reported in Fig. 2.

## Management of acute dyspnea with non-invasive respiratory support

In this section, the discussion on the management of acute dyspnea will be focused on the use of non-invasive respiratory support strategies which may be instituted by the ED physician, including high-flow nasal oxygen (HFNO), continuous positive airway pressure (CPAP) and non-invasive ventilation (NIV) [90–93]. We will not discuss the pharmacological treatment of patients with dyspnea [3, 94, 95], nor the management of acutely dyspneic patients requiring intubation and invasive ventilation at ED presentation.

Possible benefits of non-invasive respiratory support are improvement in gas exchange, amelioration of dyspnea and reduction of the work of breathing while maintaining spontaneous breathing, so to limit the requirements of sedation and to prevent endotracheal intubation and invasive ventilation [94], together with their associated risks, as airway trauma, ventilator-induced lung injury and diaphragm dysfunction [95]. In addition, in patients with acute heart failure, non-invasive ventilatory support can have favorable hemodynamic effects, reducing intrathoracic blood volume, decreasing the left ventricular afterload and increasing the cardiac output [96, 97].

Recently, it has been hypothesized that the high inspiratory drive, which is often present in spontaneously breathing patients with acute respiratory failure, may produce large intrathoracic pressure swings, generating dangerously large tidal volumes (V_T_), distorting the heterogeneous parenchyma and promoting leakage of fluid from pulmonary vessels (P-SILI, patients’ self-inflicted lung injury) [98]. If this highly controversial hypothesis [99, 100] proved true, non-invasive ventilatory support would be a double-edged sword, reducing P-SILI as long as it is able to reduce the respiratory drive and patients' inspiratory efforts, but paradoxically enhancing this risk as patients are free to maintain large intrathoracic pressure swings [101, 102]. Indeed, induction of P-SILI has been advocated as one of the causes of the increased mortality of patients who require intubation and mechanical ventilation after non-invasive support failure [103]. Irrespective of the controversy around P-SILI, these considerations stress how, as for any treatment, the success of non-invasive support is strongly affected by careful patient selection, and strict physiological monitoring remains crucial [8, 18, 94, 104]. Finally, the use of these forms of ventilation should not delay in any case the prompt institution of invasive ventilation, whenever a patient’s improvement under non-invasive support remains unsatisfactory [91, 105].

### Acute cardiogenic pulmonary edema

Since their introduction in the clinical practice, compared to conventional oxygen therapy, the use of CPAP and, subsequently, NIV have clearly demonstrated superior beneficial effects in patients with acute cardiogenic pulmonary edema in terms of several cardiopulmonary physiological variables: these include a higher reduction of respiratory rate, respiratory work and dyspnea [106–110], increase in arterial oxygenation and in the ratio between PaO_2_ and the fraction of inspired O_2_ (PaO_2_/FiO_2_) [106, 107, 111, 112], decrease in PaCO_2_ together with an increase in pH [106, 107, 110, 112, 113], decrease in heart rate, in right and left ventricular preload and in blood pressure [106, 107, 109, 110, 113]. Most data seems to agree in indicating that CPAP and NIV have similar efficacy in improving pulmonary and cardiovascular function [109, 110, 113]. However, a few studies suggest that NIV could be more effective than CPAP in unloading respiratory muscles [109] and improving circulatory stress, dyspnea and gas exchange, particularly in patients with pulmonary edema and acidosis, and in reducing time to improvement in oxygenation [108, 114, 115]. The concern regarding the possible increase in the incidence of acute myocardial infarction with NIV compared to CPAP or standard oxygen therapy, initially raised by Mehta and coll. [108], was not confirmed in larger studies [110, 113, 115–117] and in several meta-analyses [92, 114, 118–121]. These results plead for the safety of these types of non-invasive ventilatory support in patients with acute cardiogenic pulmonary edema.

The usefulness of CPAP and NIV in cardiogenic pulmonary edema has been also assessed in terms of endotracheal intubation rate, length of hospital stay and mortality, with non-univocal results. Compared to conventional oxygen therapy, a lower requirement of endotracheal intubation has been demonstrated in many studies both for CPAP and NIV [92, 106, 111, 112, 119–122]. However, other studies failed to demonstrate this benefit [111, 113, 116]. Moreover, when present, the reduction in intubation rates appears similar in both CPAP and NIV [92, 108, 110, 113, 115, 117–121]. In terms of length of hospital stay, data are not conclusive [121] but it seems that CPAP and NIV do not provide any significant reduction, both when compared to standard oxygen therapy [112, 114] or one to the other [108, 111, 115]. Evidence regarding mortality in acute pulmonary edema patients treated with non-invasive ventilatory support strategies remains partly discordant: compared to standard therapy, no improvement in mortality was reported by some studies both for CPAP [111–113, 116] and NIV [113, 114]. Indeed, the largest multicenter randomized trial comparing CPAP and NIV with standard oxygen therapy did not find any survival (and intubation) benefit [113]. It should be noted that the study was characterized by a relatively low degree of hypoxemia at baseline and a frequent crossover among groups (20% of patients), warranting caution in the interpretation of these results. However, meta-analyses conducted both before and after this study consistently demonstrated a survival benefit for both strategies, with no difference between CPAP and NIV [92, 110, 115, 118–121]. For these reasons, the recent official ERS/ATS clinical practice guidelines on non-invasive ventilation for acute respiratory failure recommended NIV or CPAP support for patients with acute respiratory failure due to cardiogenic pulmonary edema [94].

Only recently the use of HFNO has been investigated in patients with cardiogenic pulmonary edema and, to date, a limited number of studies is available. Two randomized trials found that, compared to oxygen therapy, HFNO reduced respiratory rate without any significant effect on the need for intubation or mortality [107, 123]. However, when HFNO was compared to helmet CPAP, although both systems improved the respiratory rate and PaO_2_/FiO_2_, greater improvements were observed in the helmet CPAP group, possibly related to the lower level of positive end-expiratory pressure (PEEP) actually applied by HFNO relative to CPAP [107, 124].

In light of the well-documented positive effects on the cardiopulmonary function of CPAP, its wide availability and easiness of use, and the absence of substantial difference in the outcomes compared to NIV, CPAP appears as the preferred non-invasive ventilatory support strategy in ED patients with acute respiratory failure due to cardiogenic pulmonary edema [94, 125, 126].

### Acute hypoxemic respiratory failure of non-cardiogenic origin

According to a recent meta-analysis [127], in patients with acute hypoxemic respiratory failure of non-cardiogenic origin (hypoxemic ARF), non-invasive ventilatory support strategies are associated with a lower risk of intubation (helmet and facemask NIV, HFNO) and lower mortality (helmet and facemask NIV) compared to standard oxygen therapy. The reduction in mortality with facemask NIV compared to oxygen therapy disappeared when patients with COPD and/or congestive heart failure were excluded, while persisted with helmet NIV. The level of certainty was low-moderate, and the risk of bias was deemed elevated. Extensive comparisons between the effects of the different non-invasive ventilatory support strategies were prevented by the paucity of studies related to some modalities, in particular helmet NIV, so evidence on which may be the best modality for hypoxemic ARF patients is lacking.

Due to these uncertainties and those related to the most appropriate timing for switching to invasive ventilation, the indications provided by current guidelines regarding the ventilatory support to be applied in hypoxemic ARF are few and with a low level of certainty, and their use largely rests on the clinical judgment of the experienced physician.

During the recent COVID-19 pandemic, the encouraging, despite variable, results obtained in hypoxemic ARF with non-invasive ventilatory support strategies and, even more decisively, the shortage of personnel, equipment and ventilators that impacted healthcare systems worldwide have led to a widespread increase in the use of CPAP, NIV and HFNO, both inside and outside the ICU setting, including the ED. However, results in terms of intubation rate and mortality have been highly heterogeneous and the variability among studies in terms of patient populations, disease severity, interfaces, settings and protocols used, renders discordant findings difficult to interpret [128–130]. Thus, in spite of the relatively vast literature already available, definitive recommendations in favor of one strategy over the others remain elusive.

#### Continuous positive airway pressure

CPAP has been proposed as a possible therapeutic strategy in patients with hypoxemic ARF because of the strong physiological rationale behind positive pressure application, which includes alveolar recruitment and lung expansion, shifting tidal ventilation on a more compliant part of the lung pressure–volume curve, improving gas exchange, and, possibly, limiting lung damage from cyclic opening-closure of airspaces [131].

In a multicenter randomized trial in patients with severe hypoxemic ARF from pneumonia, beside improving oxygenation, helmet CPAP reduced the need for intubation (15% vs 63%) without any difference in-hospital mortality compared to oxygen supplementation alone [132]. The improvement of oxygenation detected by Brambilla et al. is in line with previous findings of Delclaux and coll., who applied CPAP via a facemask. This group, however, was unable to demonstrate significant differences in the rate of endotracheal intubation [133], possibly because of a highly heterogeneous patient population and the use of a facemask instead of helmet.

A small study by L’Her and coll. [134], comparing the short-term effects of CPAP and NIV with two levels of support and PEEP (10–10 and 15–5 cmH_2_O, respectively) in patients with hypoxemic ARF from an acute lung injury, demonstrated that CPAP, despite improving oxygenation similarly to NIV, failed to unload respiratory muscles as NIV did. Interestingly, dyspnea was better relieved at higher support pressure.

In patients with hypoxemic ARF from COVID-19, CPAP has been demonstrated to provide better results in terms of intubation rate [135, 136], especially in patients with limitations of care (such as patients with a “do not intubate” order) [137] relative to standard oxygen therapy. However, the largest trial comparing CPAP with oxygen therapy [135] did not report any benefit in terms of mortality.

Considering the limited available evidence, further studies are needed to attribute to CPAP a precise role in the management of hypoxemic ARF: as far as respiratory muscles unloading and dyspnea reduction are concerned, CPAP is probably less effective than NIV with adequate PEEP, in spite of similar oxygenation benefits.

#### Non-invasive ventilation

The use of NIV in patients with hypoxemic ARF have been associated with a greater and faster improvement in respiratory rate and oxygenation compared to standard oxygen therapy [138, 139]. In some studies, facemask NIV shortened ICU stay [138, 140, 141] and lowered the intubation rate [138, 141], but in others did not [142, 143]. In terms of ICU mortality, no benefit was reported by many studies comparing facemask NIV with standard oxygen therapy or mechanical ventilation [138, 140–144]. A possible explanation for these discordant results is that successful NIV improves the outcome of patients by avoiding intubation and its related complications; however, intubation after NIV failure (defined as intubation either due to patient intolerance to NIV or ensuing medical indication) is associated with higher mortality rates compared to patients who are intubated without prior NIV [145, 146]. If the high mortality observed after NIV failure was due to delayed intubation, then identifying hypoxemic ARF patients at risk of NIV failure becomes important, so to treat them with invasive ventilation without delay. Predictors of NIV failure include a higher severity of illness expressed by SAPS II and APACHE II scores, shock, active cancer, lower Glasgow coma scale [144, 147–149], low PaO_2_/FiO_2_ [144, 147, 148], persistence of high inspiratory efforts [150, 151], and presence of ARDS [139, 144, 147, 152].

The setting of the support pressure and PEEP levels, together with the choice of the interface are important aspects in the management of hypoxemic ARF patients with NIV, but, at present, precise indications in this regard are unavailable. If the P-SILI hypothesis proved true, it would be convenient to set the ventilator so as to keep V_T_ in a safe range (6–8 mL/kg). However, this is difficult at best, as a respiratory drive in hypoxemic ARF patients is frequently elevated. Indeed, ~ 80% of the 66 hypoxemic ARF patients studied by Carteaux and coll. breathed during facemask NIV with a V_T_ > 8 mL/kg. Interestingly, the same Authors found that a V_T_ > 9.5 ml/kg was an independent risk factor for NIV failure [153].

NIV is not without drawbacks. Application of elevated PEEP during NIV by a facemask may be problematic, as the high pressures can increase air leaks, gastric insufflation, and patient intolerance. Additionally, prolonged application of a facemask may lead to facial skin necrosis and eye irritation [134, 154]. The delivery of NIV by helmet has been associated with less collateral effects while providing similar oxygenation improvements. Interestingly, the first randomized trial comparing facemask and helmet NIV in ARDS patients [155] showed a lower intubation rate, ventilator-free days and also 90-day mortality using helmet NIV (18% vs 61%, 28% vs 12.5% and 34.1% vs 56.4%, respectively). These impressive results are to be confirmed by future multicenter randomized trials [156]. A randomized clinical trial by Grieco and coll. [91] comparing helmet NIV followed by HFNO with HFNO only in patients with hypoxemic ARF from COVID-19 suggested that helmet NIV may be superior to HFNO, not only in terms of oxygenation benefit and dyspnea relief but also of intubation rate, despite no difference in mortality.

Overall, considering the lack of clear and conclusive evidence on the use of NIV in hypoxemic ARF, currently no guidelines recommend its use in this patient population [94, 157]. Nevertheless, the above findings suggest that NIV may help preventing intubation and the complications associated with mechanical ventilation but its use in this population requires a particularly careful patient selection, with older age, higher illness severity, more severe hypoxemia at baseline, failure to improve after one hour of treatment and moderate-severe ARDS advising against its application.

#### High-flow nasal oxygen

Recently, there has been growing interest in the use of HFNO as a non-invasive therapeutic strategy to treat patients in hypoxemic ARF. From a physiological perspective, potential beneficial effects of HFNO are reduction of nasopharyngeal resistance, dead space washout, application of a variable PEEP and enhanced oxygen delivery [158, 159]. All these factors, by improving respiratory mechanics and gas exchange, and therefore decreasing the respiratory drive, tend to decrease inspiratory efforts [160]. This reduction is, however, modest relative to the one attainable by NIV, which provides, in addition to PEEP, a support pressure [151].

As already mentioned, a non-trivial aspect that may strongly impact the probability of success of a non-invasive support strategy is the amelioration of dyspnea [161] and patient’s comfort, this latter markedly influencing treatment duration. In this regard, compared to facemask NIV, but not to helmet NIV, HFNO seems to have a better tolerability and dyspnea improvement profile [151, 162, 163].

However, large randomized trials are still needed to determine whether the physiological benefits of HFNO could translate into improvement of clinical outcomes, i.e. intubation rate and mortality. In a seminal French multicenter randomized trial [163], HFNO, relative to facemask NIV and oxygen therapy, did not significantly decrease intubation rate (38% vs 50% vs 47%, respectively) in all patients enrolled, but only in those with a PaO_2_/FiO_2_ ≤ 200 mmHg; moreover, 90-day mortality, a secondary outcome, was lower in the HFNO group. Despite promising, these results should be interpreted cautiously, as the study had low power to detect differences in intubation rate, and the reduction in mortality was based on a relatively small number of events.

Despite initial limited use due to concerns of possible enhanced viral transmission to healthcare workers, HFNO has been compared to conventional oxygen therapy in COVID-19 patients in three multicenter randomized clinical trials [135, 164, 165] with positive results: even if a reduction in mortality was not reported by any of the three, two of them showed for HFNO a lower intubation rate.

### Acute hypercapnic respiratory failure of non-cardiogenic origin

Non-invasive ventilation represents an established approach in patients presenting with hypercapnic decompensated respiratory failure of various origin [97]. Since the 80s, NIV has been widely used in different settings due to favorable data about short and long-term survival, length of ICU stay [166] and lower risk of complications such as ventilator-associated pneumonia [167] compared with invasive mechanical ventilation, especially in acute exacerbations of COPD, acute on chronic hypercapnic respiratory failure in obese patients or patients with obesity/hypoventilation syndrome and acute respiratory infections in patients with neuromuscular diseases (NMD) [168].

Acute hypercapnic respiratory failure in the context of exacerbated COPD, with or without chronic hypercapnia, represents one of the main indications for NIV. In fact, since the seminal study by Brochard and colleagues [169] demonstrating that NIV compared with standard therapy reduced the need for endotracheal intubation and improved survival reducing the length of hospital stay, numerous large randomized trials have confirmed the efficacy of NIV and sometimes its superiority to invasive mechanical ventilation, especially in terms of in-hospital complications [170]. NIV is currently suggested for patients with acute exacerbation of COPD with respiratory acidosis (pH ≤ 7.35 with a PaCO_2_ > 45 mmHg) and respiratory distress but not to prevent respiratory acidosis in those with normal pH and elevated PaCO_2_ [97]. However, there is currently no pH threshold value under which the institution of NIV can be considered. In any case, all recommendations stress that NIV should be delivered by trained staff, with adequate monitoring available and close assessment of its efficacy in terms of pH improvement must be performed to avoid delayed intubation and rapid deterioration of the patient’s clinical status. Hypercapnic coma does not represent a contraindication for the application of NIV, especially in patients with reversible hypercapnia such as in case of COPD exacerbation, but is significantly associated with improvement of consciousness one hour post-NIV initiation and with SOFA score at admission [171].

Patients with acute asthma exacerbations that develop hypercapnic respiratory failure might benefit from NIV, although its superiority compared to standard therapy in terms of endotracheal intubation and survival has not been clearly demonstrated yet. An RCT showed a faster improvement in FEV1, lower bronchodilator administration and hospitalization rate [172] in patients treated with NIV compared with standard oxygen. A study conducted in patients with severe hypercapnic respiratory failure and acidosis demonstrated that, unless needing immediate intubation for respiratory arrest, patients with status asthmaticus with mild acidosis could respond well to NIV, reducing the need for endotracheal intubation [173]. The main hypothesis on the beneficial effects of NIV in asthma and, particularly, in patients with status asthmaticus is that positive pressure might act as a stabilizer for bronchoconstricted airways and contribute to mitigate airway closure. This might explain the improvements in FEV1 and dyspnea observed with higher versus lower inspiratory pressures during mild to moderate asthma exacerbations [174].

NIV might have beneficial effects also in patients presenting with asthma-COPD overlap syndrome, but overall, considering the rapid decline of respiratory function and the fluctuation of bronchoconstriction in a patient experiencing an asthma attack, NIV might be suggested only if delivered by experienced physicians and trained ED nurses, and in those patients with a slow response to medical treatment, mild respiratory acidosis, and, obviously, in absence of need of immediate endotracheal intubation [97, 175].

NIV is the gold standard treatment for long-term respiratory support in patients with chronic hypercapnic respiratory failure secondary to neuromuscular diseases (NMD) and thoracic abnormalities [168]. However, to date, little is known about the effects of NIV compared with endotracheal intubation in patients presenting with acute respiratory failure from a rapidly progressive NMD or during an exacerbation of a slowly progressive NMD [176]. Some line of evidence suggests that patients with NMD and hypercapnic respiratory failure presenting without bulbar involvement should be assessed for an NIV trial [176], nonetheless, well-designed and stratified RCTs are currently lacking. To date, the effects of the application of NIV in patients with decompensated obesity-hypoventilation syndrome have been investigated only in a few studies, with positive results in terms of avoidance of endotracheal intubation and survival [177–181]. Yet, due to the sample size and the lack of randomization, a clear recommendation, in this case, cannot be provided.

#### High-flow nasal oxygen

While the clinical utility of HFNO in patients presenting with acute hypoxemic respiratory failure is quite established, less is known of the effects of HFNO in those with acute hypercapnic respiratory failure. Some evidence has recently shown that HFNO might represent a valid alternative to NIV in certain groups, such as patients with acute COPD exacerbations and mild hypercapnia or respiratory acidosis, being not inferior in avoiding endotracheal intubation, in improving respiratory rate and gas exchange [182–184]. However, the available studies are small, often not randomized and quite heterogeneous especially in terms of HFNO and NIV settings [185]. Consequently, further research is needed to identify the best responder to HFNO application during an episode of acute hypercapnic respiratory failure.

## Conclusions

Dyspnea is a symptom, generated by complex interactions between various physiological, psychological, pathological, and environmental factors, frequently leading patients to the ED. Its non-specificity makes the rapid and accurate identification of the underlying causes a clinical challenge. Nevertheless, dyspnea requires prompt diagnostic evaluation, as some diseases causing dyspnea can be life-threatening, and delaying diagnosis can increase morbidity, time to discharge and treatment costs. Once history and examination generate clinical suspicion for various diagnoses, they should be confirmed or disproved by the use of biomarkers and imaging techniques. Among them, lung ultrasound is gathering interest and popularity among ED physicians, and it is likely that in the future its use will become widespread, as long as more clinical studies will support its utility in the ED setting.

Even before reaching a correct diagnosis of the cause of dyspnea, ED physicians by inspection and physical examination should promptly identify patients with severe respiratory distress, that may require non-invasive ventilatory support or immediate endotracheal intubation.

## Data Availability

Not applicable.
